# Modulation of Crustacean Innate Immune Response by Amino Acids and Their Metabolites: Inferences From Other Species

**DOI:** 10.3389/fimmu.2020.574721

**Published:** 2020-11-05

**Authors:** Zishu Huang, Jude Juventus Aweya, Chunhua Zhu, Ngoc Tuan Tran, Yujian Hong, Shengkang Li, Defu Yao, Yueling Zhang

**Affiliations:** ^1^ Institute of Marine Sciences and Guangdong Provincial Key Laboratory of Marine Biotechnology, Shantou University, Shantou, China; ^2^ Shantou University-Universiti Malaysia Terengganu (STU-UMT) Joint Shellfish Research Laboratory, Shantou University, Shantou, China; ^3^ College of Fisheries, Guangdong Ocean University, Zhanjiang, China; ^4^ Guangdong Yuequn Marine Biological Research and Development Co., Ltd., Jieyang, China

**Keywords:** crustaceans, amino acids, metabolism, immune-modulation, innate immunity

## Abstract

Aquaculture production of crustaceans (mainly shrimp and crabs) has expanded globally, but disease outbreaks and pathogenic infections have hampered production in the last two decades. As invertebrates, crustaceans lack an adaptive immune system and mainly defend and protect themselves using their innate immune system. The immune system derives energy and metabolites from nutrients, with amino acids constituting one such source. A growing number of studies have shown that amino acids and their metabolites are involved in the activation, synthesis, proliferation, and differentiation of immune cells, as well as in the activation of immune related signaling pathways, reduction of inflammatory response and regulation of oxidative stress. Key enzymes in amino acid metabolism have also been implicated in the regulation of the immune system. Here, we reviewed the role played by amino acids and their metabolites in immune-modulation in crustaceans. Information is inferred from mammals and fish where none exists for crustaceans. Research themes are identified and the relevant research gaps highlighted for further studies.

## Introduction

Crustaceans constitute an important part of the marine ecosystem, with shrimp and crabs forming a large proportion of aquatic food destined for human consumption. Over the past decade, shrimp and crab farming have expanded rapidly bringing with huge economic benefits (1). As invertebrates, crustaceans depend solely on innate immune response for defense and protection against pathogens (2, 3). The innate immune system is constituted by cellular and humoral immune responses (4). The cellular immune response mostly takes place in hemocytes where a variety of pattern recognition receptors (PRR) on cell membranes detect and eliminate pathogens *via* phagocytosis, apoptosis, nodule formation and encapsulation (5). On the other hand, the humoral immune response mainly depends on immune factors such as prophenoloxidase (proPO), lectins, antimicrobial peptides (AMP), etc., found in the hemolymph (6–8).

When the immune system is activated its demand for energy and metabolic substrates increases substantially. The immune system derives these metabolic substrates mainly from nutrients to provide energy as well as serve as precursors for the synthesis of new cells, effectors (e.g., antibodies, cytokines, and acute phase proteins) and protective molecules (e.g., glutathione) (9). There is therefore a close link between the immune and metabolic systems. Among all metabolic substrates required by the immune system, amino acids and their metabolites have attracted much interest (10, 11), probably due to their diverse functions and effect on several physiological and pathophysiological processes. For instance, arginine (Arg) can be converted to citrulline and nitric oxide (NO) under the action of nitric oxide synthase (NOS), with NO functioning as an effector molecule of tumor and microbial immunity, as well as a regulator of many immune cells (12). Arg is also catabolized to polyamines under the action of arginase and ornithine decarboxylase (ODC). In mammals, inhibition of ODC has been used in what is termed polyamine-blocking therapy (PBT), a strategy that combines the inhibition of polyamine biosynthesis with the simultaneous blockade of polyamine transport to enhance anti-tumor immune response (13).

In crustaceans, amino acids and their metabolites play important roles in several physiological and pathophysiological processes including innate immune response. For instance, supplemented dietary Arg has been shown to improve the body weight and growth rate of juvenile kuruma shrimp (*Penaeus japonicus*) (14), while tryptophan (Trp) is capable of reducing the aggressive behavior of juvenile mud crab (*Scylla serrata*) as well as enhances their anti-stress ability (15). Although amino acids and their products are reported to affect the growth and immune indices of crustaceans (16–19), very few studies have explored their involvement in immune response and the molecular mechanisms involved. In any case, amino acids and/or their metabolites seem to play pivotal immune-metabolic regulatory roles in crustaceans. This review therefore brings together important findings on the effects of amino acids and their metabolites on the immune system of crustaceans, in most cases drawing inferences from other species where such information does not exist for crustaceans. Research areas are also identified with the hope that when these are further explored, it could lead to a better understanding of the role played by amino acids and their metabolites in immune-metabolic regulation in crustaceans.

## Amino Acid Metabolism and Crustacean Immunity

As the main building blocks of proteins, amino acids are important in the growth and development of animals. Recent studies have also shown that amino acids and their metabolites play an important role in the immune system. For instance, in mammals, Arg is involved in the regulation of immune cells proliferation, by modulating the levels of NO, which consequently affect the immune system (20). Although few studies have explored the role of amino acids metabolism (metabolites) in crustacean immunity, an increasing number of studies have reported that the metabolism and/or metabolites of Arg, Trp, lysine (Lys), methionine and cysteine play key important roles in immune response in crustaceans, as in other marine species (**Table 1**).

**Table 1:** Role of amino acids and their metabolism in the immune response of crustaceans and other marine species.

Amino acid	Species	Factor/immune response	Reference
Arginine	*Penaeus vannamei*; *Eriocheir sinensis*	Dietary Arg improves antioxidant enzyme activity and immune response	(21, 22)
	*P. vannamei*	Increased NO and NOS mRNA levels to improve antibacterial immune response	(23)
	*P. vannamei*; *Panulirus argus*	Increased NO levels and NOS activity to improve antibacterial immune response	(24, 25)
	*Penaeus japonicus*	Decreased arginine kinase activity attenuates WSSV replication	(26)
	*Paphia malabarica*	Increased iNOS activity as an antibacterial immune response	(27)
	*Megalobrama amblycephala*	Dietary Arg improves antioxidant capacity and immune response	(28)
Tryptophan	*E. sinensis*	Dietary Trp increases dominant intestinal bacteria abundance, serum CAT and AKP activity, and improves immune response	(29)
	*E. sinensis*	Dietary Trp increases THC, hemocyanin, ACP and ALP activity, and hemocyte phagocytic activity	(30)
	*E. sinensis*	Melatonin (injected) increases THC, hemocyanin, and activity of ACP and GSH-Px	(31)
	*E. sinensis*	Melatonin restores oxidative damage, stabilizes ACP, AKP, and Na^+^-K^+^-ATPase activity, increase Cyt-C content, restores apoptotic rate and phagocytic activity of hemocytes	(32)
	*E. sinensis*	Melatonin (injected) increases SOD activity and decrease MDA content to enhance antioxidant capacity	(33)
	*Dicentrarchus labrax*	Increase Trp levels decrease inflammatory response *via* immunosuppression	(34)
Lysine	*Astacus leptodactylus leptodactylus*	L -carnitine improves antioxidant defense by increasing activities of PO, SOD, GSH and GPX	(35)
	*Apostichopus japonicus*	Increased Lys enhances CAT and AKP activity to improve antioxidant and immune response	(36)
	*Ctenopharyngodon idella*	Dietary Lys increases SOD, GPX and Nrf2 levels to improve lipid and protein oxidation	(37)
	*Acanthopagrus schlegelii*	Dietary carnitine increases LZM and CAT activity, but inhibits expression of pro-inflammatory factors	(38)
	*Rhynchocypris lagowski*	Carnitine reduces inflammatory response by Nrf2/Keap1 activation to inhibit NF-κB signaling pathway	(39)
Methionine and cysteine	*Oreochromis niloticus; D. labrax*	Dietary Met increases C3 and C4 levels, CAT, GPX, and LZM activity to enhance immune response and antioxidant capacity	(40, 41)
	Dicentrarchus labrax	Met promotes immune cells proliferation by regulating polyamines synthesis	(42)
	*D. labrax*	Met enhances leukocytes proliferation and reduces expression of pro-inflammatory genes	(43)
	*E. sinensis*	GSH supplementation promotes expression of immune genes (*alf1* *alf2* *alf3*, *crus1*, and *crus2*),,	(44)
	*E. sinensis*	Dietary GSH increases SOD, GPX and GST activity to resist oxidative stress. Also reduces apoptosis by inhibiting expression of *caspase-3*, *caspase-8*, and *caspase-9*	(45)
	*P. vannamei*	Dietary GSH increases ACP, AKP and SOD activity, and sensitivity to *V. alginolyticus* infection	(46)
	*E. sinensis*	Taurine supplementation increases expression of immune genes and AMPs	(47)
Branched chain amino acid	*M. amblycephala*	Leu increases antioxidant enzyme activity and the levels of C3 and IgM	(48)
	*Labeo rohita*	Leu increases expression of LZM, C3, β-microglobulin, IgM, SOD, GPx, Nrf2, NKF-β, and TLR22, and decreases TNF-β, Keap1, and IL-1B	(49)
	*Paralichthys olivaceus*	Ile enhance respiratory burst and total Ig content	(50)
	*Trachinotus ovatus*	Val increases LZM activity and levels of C3, C4, and IgM	(51)
	*Portunus trituberculatus*	Dietary Leu improves antioxidant capacity by increasing PO and SOD activity	(52)
Glutamate and glutamine	*Cyprinus carpio* var. Jian	Glu supplementation induces Nrf2 to enhance antioxidant enzymes activity	(53)
	*Oreochromis niloticus*	Gln supplementation improves macrophages phagocytosis and bactericidal ability. Promote lymphocyte proliferation	(54)
	*Oncorhynchus mykiss*	Gln increases number of B-lymphocytes and secretion of Igs through NODs signaling pathway	(55)
	*P. vannamei*	Glu-driven anaplerosis provides ATP and lipids for WSSV replication	(56, 57)
Phenylalanine	*Danio rerio*	Phe helps clear drug-resistant bacteria (e.g. *Vibrio alginoyticus*), through an unknown pathway	(58)
	*Oreochromis niloticus × Oreochromis aureus*	Dietary Phe increases LZM and CAT activity	(59)
	*C. idella*	Phe supplementation increases expression of intestinal IL-10, TGF-β1, TOR, IκBα, and Nrf2	(60)
Tyrosine	*P. vannamei*	Tyrosine hydroxylase knockdown enhances immune response and delays the decreased immune response under low temperature stress	(61, 62)
	*Macrobrachium rosenbergii*	DA (injected) suppresses immune response and increases susceptibility to *Lactococcus garvieae* infection	(63)
	*Penaeus monodon*	DA (injected) suppresses immune response and increases susceptibility to *Photobacterium damsela* infection	(64)
	*P. vannamei*	DA receptor coupling with G protein activates the CAMP- PKA, DAG-PKC, or CAM pathway to regulate immune response	(65)
Proline	*P. vannamei*	Pro supplementation improves antioxidant and immune capacity	(66)
Histidine	*M. amblycephala*	Dietary His inhibits nuclear import of Nrf2 and decreases expression of antioxidant enzymes	(67)
	*C. idella*	His deficiency/excess cause oxidative damage, increases pro-inflammatory factors and decreases anti-inflammatory factors expression	(68)
	*E. sinensis*	Histamine increases PO and SOD activity but decreases levels of THC, ACP, and AKP	(69)
Threonine	*C. idella*	Thr deficiency decreases LZM and ACP activity, and levels of C3, C4, and IgM. Decreases expression of AMPs	(70)
	*M. amblycephala*	Thr supplementation increases levels of C3, C4 and IgM, and activity of SOD, CAT, and GPX	(71)
	*M. amblycephala*	Excess or deficient Thr causes damage to antioxidant and immune systems	(72)
Glycine	*C. idella*	Gly and N-acetyl cysteine (NAC) supplementation improves antioxidant capacity	(73)

ACP, acid phosphatase; AMPs, antimicrobial peptides; HSP, heat shock proteins; NO, nitric oxide; IL, interleukin; TNF-α, tumor necrosis factor-α; IFN-γ, interferon-γ; IDO, Indoleamine 2, 3-dioxygenase; CAT, catalase; Cyt-C, cytochrome C; GPx, glutathione peroxidase; GSH, glutathione; GR, glutathione reductase; Ig, immunoglobulin; IgG, immunoglobulin G; BCL2, B-cell lymphoma 2; mTOR, mechanistic target of rapamycin; RPS6KB1, ribosomal protein S6 kinase B1; IgM, immunoglobulin M; C3, component 3; SOD, superoxide dismutase; MDA, malondialdehyde; Nrf2, nuclear factor erythroid 2-related factor 2; NKEF-β, natural killer-cell enhancing factor β; TLR22, toll-like receptor-22; Keap1, Kelch-like-ECH-associated protein 1; GPCRs, G protein-coupled receptors; ERK, extracellular regulated protein kinases; LZM, lysozyme; C4, complement 4; MDSCs, myeloid-derived suppressor cells; TGF-β1, transforming growth factor-β1; IκBα, inhibitor of nuclear factor κBα; DAO, diamine oxidase; THP-1, the human monocytic leukemia cell line; ICAM-1, intracellular adhesion molecule-1; NF-κB, nuclear factor-κB; PBMCs, peripheral blood mononuclear cells; TIBC, total iron-binding capacity; Muc2, Mucin-2; GSH-Px, glutathione peroxidase activity; NOS, nitric oxide synthase.

### Arginine

Arg is one of the most versatile amino acids that can be converted into other amino acids (proline, glutamic acid, and glutamine) or metabolized to urea *via* the urea cycle, as well as to polyamines, NO, creatine, and other essential non-protein substances (12). Arg is therefore an important precursor that generate metabolites and intermediates vital for the immune system. In the metabolism of Arg, arginase and NOS are two key enzymes. Arginase catalyzes the catabolism of Arg into urea and ornithine, which then generate polyamines by the action of ODC (74). Polyamines are important Arg metabolites that have antitumor effect in mammals (75, 76) and are also involved in the synthesis of T-cells (77). Similarly, NO, which is generated from Arg metabolism by the action of NOS, is an effective antibacterial agent against intracellular and extracellular pathogens (24, 78). While NO has antimicrobial effects and also modulates immune response in host cells, excess NO levels could promote peroxynitrite synthesis to generate hydroxyl radicals that cause cell damage and/or cell death (20, 79).

Optimum proportions and levels of amino acids are required by crustaceans for proper growth and physiological/metabolic functions, although differences exist among different species. The Pacific White shrimp *Penaeus vannamei* requires 4.77% optimum level of dietary Arg (21), while 5.47% is required by *Penaeus monodon* (80). Supplemented dietary Arg is reported to affect the metabolic activity of crustaceans by regulating the activity of some enzymes. For instance, aspartate aminotransferase (AST) and alanine aminotransferase (ALT), which are key enzymes in amino acids metabolism, are important indicators of hepatopancreas function in shrimp (81, 82). The serum levels of AST and ALT increased significantly when juvenile swimming crab (*Portunus trituberculatus*) were fed on low Arg diets (82). Arg supplementation also increases the activities of antioxidant enzymes and immune-related enzymes, thereby improving the antioxidant capacity, immunity, and disease resistance of juvenile *P. vannamei* and *E. sinensis* (21, 22).

Some Arg metabolic pathway enzymes in crustaceans are reported to modulate antimicrobial immune response. When the Caribbean spiny lobster (*Panulirus argus*) and red swamp crayfish (*Procambarus clarkii*) were challenged with *Escherichia coli* or lipopolysaccharide (LPS), NO levels and the activity of NOS both increased, as part of the immune response (25, 83). It has also been observed that when an NOS inhibitor or *P. argus* generated anti-NOS serum is administered to *P. vannamei*, it reduces bacterial clearance, further illustrating the importance of NOS in the immune defense of crustaceans (24, 83).

**Figure 1:**
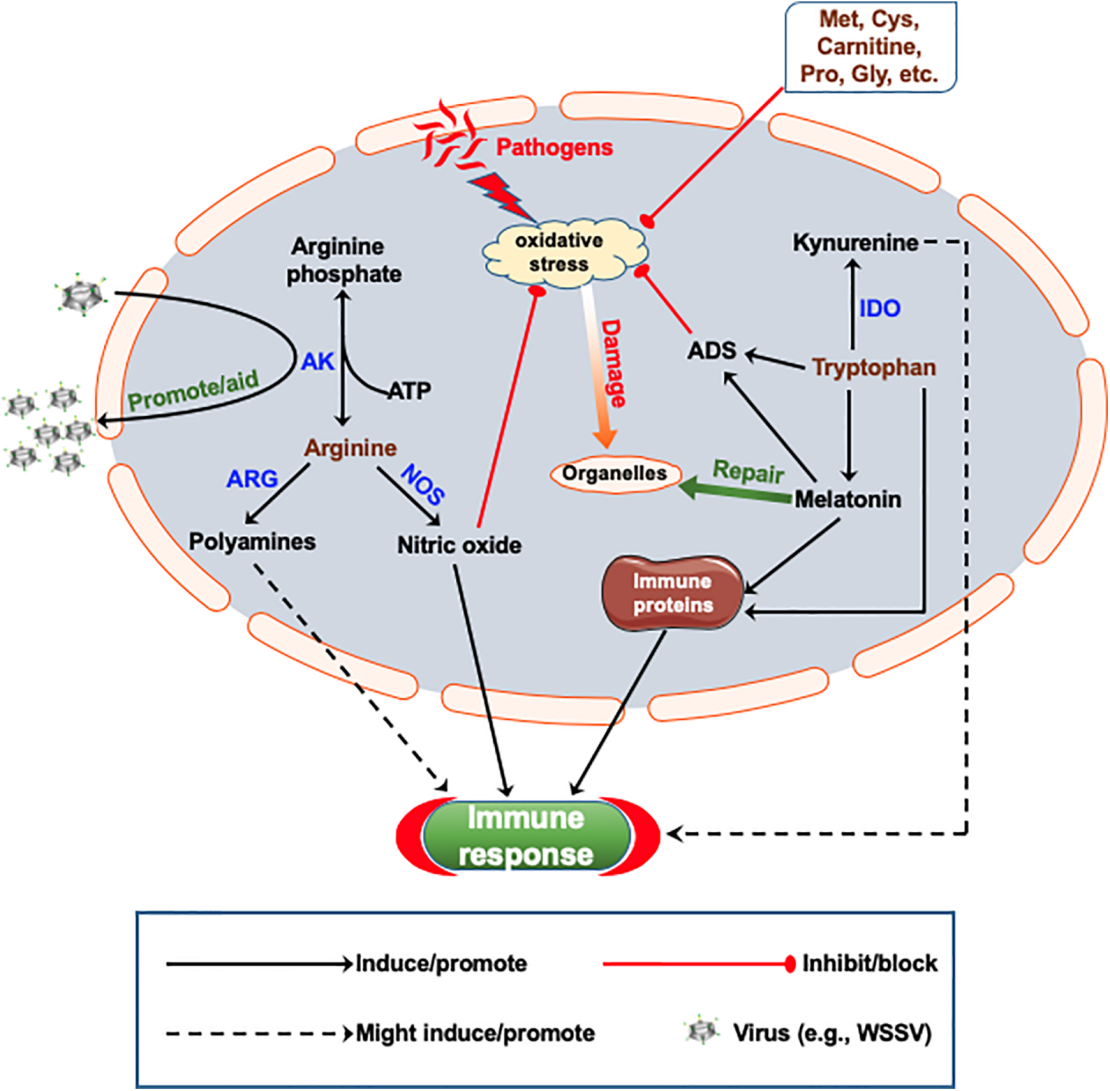
Proposed schematic representation of how amino acids metabolism and immune-modulation occur in crustaceans. Nitric oxide (NO) is generated from arginine (Arg) by the action of nitric oxide synthase (NOS), which counteracts pathogen-induced oxidative stress and promote immune response. Polyamines, as downstream products of Arg metabolism, promote immune response, while arginine kinase (AK) can catalyze the conversion of Arg to arginine phosphate, which can be coopted to promote replication of white spot syndrome virus (WSSV). Tryptophan (Trp) and its metabolite melatonin can activate the antioxidant system (ADS) and promote the expression of immune proteins as well as counteract oxidative stress. Melatonin also promotes the repair of oxidative stress induced organelle damage. Trp could also suppress inflammatory response through the kynurenine pathway. Some amino acids such as Met, Cys, Pro, Gly, etc., have direct antioxidant activity due to their chemical properties. IDO, Indoleamine‐2,3‐dioxygenase; ARG, Arginase; Met, methionine; Cys, cysteine; Pro, proline; Gly, glycine.

Arg kinase is one of the most important enzymes that regulates energy metabolism in invertebrates (84). Invertebrates mainly store their energy in the form of Arg phosphate, which is converted to ATP and Arg by the action of Arg kinase during energy (ATP) demand, while under ATP saturation, Arg kinase catalyzes the synthesis of Arg phosphate as energy store (85). This could be one of the reasons why in crustacean Arg kinase levels change in response to immune stimulation and virus infection (84, 86, 87). In *P. japonicus*, the Arg kinase homolog *Mj*AK is reported to promote WSSV replication while the Cdc42 homolog *Mj*Cdc42, inhibits WSSV replication by interacting with the active site of *Mj*AK to inhibit its enzyme activity (26). Levels of Arg and Arg metabolites as well as the activity of Arg kinase therefore affect energy homeostasis in crustaceans, which consequently affect the immune system (**Figure 1**).

### Tryptophan

The aromatic amino acid, Trp, is an essential amino acid in most animals (including crustaceans) that must be obtained from diets, as it cannot be *de novo* synthesized. In addition to its role as a protein building block, Trp is metabolized into other active substances *via* two main pathways, i.e., (i) conversion to 5-hydroxytryptophan under the action of Trp hydroxylase, which is decarboxylated to serotonin (5-HT), and then finally to melatonin (MT) by N-acetyltransferase, (ii) Trp is also metabolized to kynurenine, catalyzed by Trp 2, 3-dioxygenase (TDO), and indoleamine 2, 3-dioxygenase (IDO), and then to acetyl-CoA and nicotinamide adenine dinucleotide (NAD+) (88, 89). Thus, Trp and its metabolites play important roles in many metabolic, physiological, and pathophysiological functions including immunity (90–93).

Most core metabolic reactions are conserved across many organisms, highlighting the fundamental role of metabolism [reviewed by (94)]. Thus, due to the paucity of information on the role of metabolic pathway components in some species, comparative studies or inferences are drawn from other organisms. For Trp metabolism, numerous mammalian studies have shown its involvement in diverse immune-related functions. For instance, Trp affects the gut immunity of piglets and mice by decreasing the expression of cytokines (e.g., TNF-α, IL-6, IL-1β, IL-17, etc.) and inducing the expression of proapoptotic caspase-8 and Bax (95). Trp also activates the aryl hydrocarbon receptor (AhR) transcription factor (96), a key regulator of immunity and inflammation in mammals (97), therefore essential for maintaining intestinal immunity and barrier function (98, 99). Despite these beneficial effects of Trp, high levels of dietary Trp could adversely affect the morphology of intestinal epithelium and tight junction proteins (100). Key enzymes in the Trp metabolic pathway have also been implicated in immune regulation. It has been reported that IDO and AhR work together to link microbial Trp catabolism and host Trp metabolites to regulate T-cells function in intestine, especially in T-cells immunity that depends on AhR [see recent review by (101)]. During infection of humans by the parasite *Toxoplasma gondii*, host cells synthesize Interferon-γ (IFN-γ) to activate IDO, so as to degrade Trp, and therefore prevent *T. gondii* replication (102, 103). The Trp metabolite serotonin (5-HT) has also been implicated in immune response in mammals, as intestinal microbiota are able to modulate host immune response by altering levels of 5-HT in models of mucosal infections (104, 105), thereby attenuating the ability to mount immune response to disease pathogens (106–108).

There is no comprehensive information on Trp metabolism in marine species as compared to terrestrial mammals, especially the role Trp plays in immune-related functions. Nonetheless, Trp and its metabolites are reported to play several physiological and immune-related functions in marine animals. In crustaceans, dietary Trp supplementation improves the growth index and survival rate (29, 109). It has also been shown that Trp (metabolites) decreases the aggressive behavior (fights/attacks) of juvenile *S. serrata* (15, 110), improves reproduction in *P. monodon* (111) and freshwater crab (*Barytelphusa guerini*) (109), as well as embryonic development of the giant freshwater prawn *Macrobrachium rosenbergii* (112). In terms of immunity, Trp supplementation increases intestinal microbiota of *E. sinensis*, resulting in higher survival rates upon bacterial challenge (29). While loss of limbs in crabs decreases their immunity and survival rate (113, 114), dietary Trp supplementation improves their immune indices [e.g., the total hemocyte count (THC), phagocytosis rate, acid phosphatase (ACP), and alkaline phosphatase (ALP) activity, etc.] as well as antioxidant capacity (30). The Trp metabolite melatonin, affects the immune system and antioxidant defense system (ADS) of crustaceans (33, 115). When eyestalk-ablated *E. sinensis* were injected with melatonin, both their immune and antioxidant capacity were enhanced in terms of increased THC and hemocyanin levels, coupled with an elevation in the activities of ALP, ACP, superoxide dismutase (SOD), glutathione peroxidase (GSH-Px), and other antioxidant enzymes (31). Melatonin injection also enhances response to oxidative damage and elimination of damaged mitochondria and hemocytes due to external stress in *E. sinensis* (32). There are however differences in the effect of melatonin in various tissues, as it does not exert direct effect on the ADS of gills in the estuarine crab (*Neohelice granulata*) but enhances that of muscle by increasing glutathione (GSH) content and glutamate cysteine ligase (γ- GCL) activity (116, 117). Although increased Trp levels tend to decrease the inflammatory response in fish (*Dicentrarchus labrax*) due to immunosuppression by Trp metabolism (34), there is dearth of information as to whether elevated levels of Trp and/or its metabolism have similar immunosuppressive effect in crustaceans. In any case, enough evidence points to the fact that Trp and its metabolites are involved in different aspects of immune regulation in crustaceans (**Figure 1**), although details of the immune-metabolic mechanisms remain unknown. More studies are needed to further explore the molecular mechanisms of immunomodulation by Trp and its metabolites in crustaceans.

### Lysine

Most animals must obtain Lys from their diets as it is an essential amino acid, which cannot be synthesized by the body. In mammals, L-lysine can be irreversibly converted into glutamate and α-aminoadipic acid through glycolysis, before being further deaminated and oxidized (118). In addition to protein synthesis, Lys can combine with methionine to form carnitine (38), which is involved in the transfer of long-chain fatty acid acyl groups to mitochondria for β-oxidation (119). The optimal dietary intake of Lys differs in different marine species and also exerts different effects on physiological and biochemical indices (36, 120–124). For example, in fish, the daily optimal Lys requirement for juvenile dusky kob or Giant kob (*Argyrosomus japonicus*) is 7.35% of the diet (125), while that of juvenile silver perch (*Bidyanus bidyanus*) is 5.96% (126). It has been observed that within a certain range, increasing Lys intake increases body weight and specific growth rate (SGR) of *Totoaba macdonaldi* (123), while excessive Lys intake affects growth and feed utilization in large yellow croaker (*Pseudosciaena crocea*; Richardson, 1846) (127). On the other hand, lack of Lys in *B. bidyanus* reduces the crude protein content of whole body, muscle and liver, but increases fat content (126).

Varying optimal dietary amounts of Lys have also been reported in different crustaceans. In juvenile Atlantic ditch shrimp (*Palaemonetes varians*), optimal Lys levels of 2.42%–2.63% have been reported (128), with 1.64% reported for *P. vannamei* (18), while 2.17% has been reported for juvenile *P. trituberculatus* (16). In *M. rosenbergii*, increased levels of Lys affect Arg retention, which suggests some antagonism between Arg and Lys levels (129), probably because they are both absorbed *via* the same brush border membrane carrier (130). Dietary Lys supplementation affect various physiological and biochemical indices in crustaceans including SGR, weight gain (WG), feed efficiency, protein efficiency ratio, protein deposition ratio, as well as AST and ALT activities (127). The activities of pepsin, trypsin and other digestive enzymes are also reported to increase upon adding the appropriate levels of Arg and Lys to diets of *M. rosenbergii* (129), while dietary Lys affects intestinal protease levels and activity of ALP in juvenile sea cucumber (*Apostichopus japonicus*) (36).

There is no direct evidence of the involvement of Lys in immune regulation in crustaceans. However, in many species, some amino acids, their metabolites as well as enzymes involved in their metabolic pathways have been directly or indirectly implicated in immune regulation [see review by (131) (Wu and Meininger, 2002)]. For instance, dietary carnitine (Lys metabolite) has been shown to increase the activity of lysozyme (LZM) and catalase (CAT) in serum and liver of juvenile black seabream (*Acanthopagrus schlegelii*) as well as increase the antioxidant capacity, but reduces inflammatory response (38, 39, 132). In crustacean, dietary supplementation of carnitine improves growth, increase feed utilization and the antioxidant system in juvenile narrow clawed crayfish (*Astacus leptodactylus leptodactylus*; Eschscholtz, 1823) (35). Most of the research on the involvement of carnitine in immune regulation in marine animals has mainly been in fish (133–135), with none on crustaceans. It is believed that carnitine regulates nutrition metabolism to enhance anti-stress response, as dietary carnitine increases lipids utilization rate to produce more energy and reduce amino acids catabolism, thereby reducing lipids peroxidation and promoting protein synthesis (136). Although few studies have explored the molecular mechanisms involved in Lys metabolism in crustacean, given that most core metabolic reactions are conserved across many organisms (94), coupled with the importance of Lys in immuno-regulation and its antagonism with Arg, the role of Lys in crustacean immunity could be inferred from other species. In any case, specific studies in crustacean are needed to explore the immune-metabolic functions of Lys, especially the involvement of Lys in the Arg-NO pathway or other immune-related pathways.

### Methionine and Cysteine

Methionine (Met) and cysteine (Cys) are two sulfur-containing amino acids that are involved in various metabolic pathways and affect several biological functions. There are three main pathways through which Met is metabolized including (i) protein synthesis; (ii) conversion to S-adenosylmethionine (SAM), an important methyl donor in the formation of polyamines, or trans-methylation of SAM to S-adenosylhomocysteine (SAH), which is further hydrolyzed to homocysteine (Hcy). Hcy can be remethylated to Met by Betaine-homocysteine methyltransferase (BHMT) and Methyltetrahydrofolate-homocysteine methyltransferase (MS); (iii) irreversible conversion of Hcy to cystathionine through the trans-sulfhydryl reaction, and then further to cysteine (137, 138). Met and its metabolites possess antioxidant capacity, as Met residues are very sensitive to oxidation and can inactivate reactive oxygen species (ROS) (139, 140). Thus, Met acts as an antioxidant to protect proteins and other macromolecules from oxidative damage (141, 142).

The involvement of Met metabolism in immune-related functions have been extensively studied in mammals, which means that inferences could be drawn from such studies to explain similar phenomenon in crustacean, given that core metabolic pathways are conserved across species. In mammals, high dietary Met content improves IgG levels and percentage of lymphocytes, as well as pathological changes due to infectious bursal disease (143). As an important immune organ in birds, bursa plays a pivotal role in immunity, hence, lack of Met inhibits the development of the bursa of Fabricius, which affects the humoral immunity of chicken (144). Dietary Met supplementation also affects the expression of inflammation-related genes (145, 146), and impact positively on gut immunity in mammals (147–149). Similarly, the addition of Met dipeptides to feed reduces the harmful effects of *Eimeria* spp. on the gut of broilers (150), while lack of Met in feed could attenuate WG, intestinal development and intestinal mucosal immunity in broilers and pigs (151, 152), or reduce resistance to parasites in rats (153).

A growing number of studies have explored the effects of dietary Met supplementation on immune-metabolic modulation in marine animals including crustaceans. Most of these studies have, however, been focused on fish. For instance, dietary Met supplements have been shown to improve growth performance in different fish species (144, 154–158). Specifically, lack of Met in feed could affect protein synthesis and reduce feed utilization in flatfish (*Solea senegalensis*) and white bass (*Morone chrysops*) (159, 160), as well as induce general mitochondrial dysfunction in liver of rainbow trout (*Oncorhynchus mykiss*) (161). On the contrary, excess dietary Met does not result in a corresponding improvement in growth rate in *O. mykiss* or orange-spotted grouper (*Epinephelus coioides*) (156, 162), and could even decrease growth rate in *P. crocea* (157). In the European sea bass *D. labrax*, dietary Met increases the number of immune cells upon immune stimulation (34), as Met is used as a precursor for the synthesis of polyamines, which is required for the regulation of immune cells proliferation (42). Dietary Met also improves immunity, by increasing leukocyte proliferation but decreases the pro-inflammatory index in *D. labrax* (43), juvenile yellow catfish (*Pelteobagrus fulvidraco*) (163) and juvenile Nile tilapia (*Oreochromis niloticus*) (40). Similarly, Met levels can potentiate the activity of SOD in juvenile *O. niloticus* (40), and increase the activities of CAT and GSH-Px in juvenile *D. labrax* (41). Despite the very few number of studies that have reported on the involvement of Met in crustacean immunity, dietary Met supplementation has been shown to improve growth performance in shrimp (*P. vannamei* and *P. monodon*) (164, 165). The question then is, does Met and its metabolites also improve the immune response and antioxidant ability in crustaceans, as observed in fish and other species? This remains an open question.

Cysteine possess special chemical properties that makes it easily oxidized like Met (166, 167), for which reason it is often used as an indicator of oxidative damage (168). The detailed metabolic pathway and functions of Cys and/or its metabolites in immune-metabolic modulation in crustaceans have not been well elucidated as in mammals, although they could be similar due to conserved core metabolic pathways across species. For instance, in mammals such as piglets, Cys is reported to improve the intestinal inflammatory response induced by dextran sodium sulfate (DSS) (169), due to the ability of Cys to decrease intestinal oxidative stress (147). Dietary Cys is reported to increase the expression of proliferating cell nuclear antigen, occludin and claudin-1 after LPS stimulation in weaned piglets, because Cys is able to protect intestinal integrity (170). While Cys is required for T-cells activation in mammals (88), T-cells are unable to convert Met into Cys, and do not have the cystine transporter to transfer cystine, which means that Cys must be provided to T-cells by antigen-presenting cells (APC) (171). Thus, in the absence of Cys, T-cells activation is attenuated and the synthesis of glutathione and DNA in cells is blocked, which eventually result in functional damage and apoptosis (172–174). During Cys metabolism, it can also combine with glutamate and glycine to form the tripeptide glutathione (GSH) in a two-step reaction catalyzed by γ-l-glutamyl-l-cysteine:glycine ligase and glutathione synthetase (175). As an important antioxidant that scavenge free radicals and ROS (176) to prevent oxidative stress, GSH has been implicated in many cellular reactions including immune regulation. In mammals, GSH is required for lymphocyte proliferation, T-cells activation and cytokine production (177–179). In addition, GSH inhibits inflammatory response (180), as well as enhance innate and adaptive immunity in humans by providing protection against microbial infection (181). Mammals also metabolize Cys to taurine or ethanesulfonic acid, one of the most abundant amino acids in cells (172) (Grimble, 2006), which plays a key role in immunomodulation (182, 183).

Limited number of studies have explored the role of Cys and its metabolites in immune-metabolic modulation in crustaceans. Nonetheless, it has been shown that dietary Cys and Met supplementation improves survival rate, feed intake, and food conversion rate in *P. vannamei* (154). In the kuruma shrimp *P. japonicus*, Cys and GSH have been shown to improve growth, inhibit phenoloxidase (PO) activity, and reduce browning due to o-quinones (184). Similarly, dietary GSH supplementation in Chinese mitten crab (*E. sinensis*), increased the levels of total protein, albumin (alB) and globulin (glB) in hemolymph, upregulated the expression of immune-related genes (44), and reduced LPS-induced pathological damage to the hepatopancreas, as well as decreased ROS levels and apoptosis (45). Dietary GSH supplementation in *P. vannamei* has also been shown to increase the activity of ACP, AKP, and SOD, while decreasing shrimp susceptible to *V. alginolyticus* infection (46). In crustaceans, taurine has been shown to improve growth and immunity by increasing body weight, SGR, expression of intestinal immune genes and antimicrobial peptides in *E. sinensis* (47), and improve the growth performance of *P. vannamei* (185). In spite of the fact that no specific studies have so far explored the role of Cys and Met or their metabolites in crustacean immunity, there are enough evidence to suggest that these amino acids play key roles in crustacean immunity. Further research should therefore explore how Cys and Met or their metabolites modulate crustacean immune response and the molecular mechanisms involved.

### Other Amino Acids

The branched chain amino acids (BCAA) leucine, isoleucine and valine, which are essential amino acids in animals (186), are important for growth, development and immunity (187, 188). Leucine (Leu) acts as a nutrient signal that regulates T-cells by activating mTORC1 (189). The mTOR signaling pathway is important for immune response, cell metabolism and other biochemical reactions (190), as it receives and organizes signals from the surrounding environment to direct T-cells differentiation and function in mammals (191). Isoleucine (Ile) is associated with intestinal immunity and can activate G-protein coupled receptors (GPCR) and extra-cellular signal-regulated kinase (ERK) signaling pathways, thereby increasing the expression of human β-defensin-2 (HBD2) (192), an important antimicrobial peptide involved in gut innate immunity (193). In fish, such as grass carp (*Ctenopharyngodon idella*), increase dietary Leu content within a certain range results in an increase in the mRNA level of Nrf2 [nuclear factor erythroid 2-related factor 2 (Nrf2)] in muscle that also shows a positive correlation with the expression of antioxidant enzymes, indicating that Leu enhances antioxidant capacity by increasing the activity of antioxidant enzymes (194). Increasing levels of Leu supplements also improves the immune capacity of juvenile blunt snout bream *(Megalobrama amblycephala*) by elevating the levels of complement component 3 (C3) and IgM (48). The effects of BCAAs on immune function has also been shown in juvenile golden pompano (*Trachinotus ovatus*) (51), juvenile olive flounder (*Paralichthys olivaceus*) (50), and *Labeo rohita* fingerlings (49). For crustaceans, dietary Leu intake has been reported to improve growth performance (195), and antioxidant capacity by increasing the activity of PO and SOD in juvenile *P. trituberculatus* (52). While the effects of Leu on the antioxidant system and the molecular mechanism involved have not been reported in crustaceans, it could be similar to that of fish or other marine species due to synonymous metabolic pathways found in different species. Further studies could explore this aspect of Leu’s role in crustacean immune response.

Glutamine (Gln) is the most abundant free amino acid in mammals (196), and plays a key role in immune response by supporting T-cells proliferation (197), macrophage development (198) and intestinal immunity (199). In marine animals, Gln supplementation can improve the antioxidant capacity of juvenile gilthead sea bream (*Sparus aurata*) (200) and enhance macrophages phagocytosis and bactericidal ability in *O. niloticus* (54). Similarly, in *O. mykiss*, Gln is reported to inhibit LPS-induced inflammatory response through the NOD signaling pathway (55). Very few studies have so far explored Gln metabolism and its metabolites on crustacean immunity. In a recent study, it has been revealed that a different type of anaplerosis exist in *P. vannamei*, where WSSV-infected cells were more likely to ingest glutamate than glutamine, as the virus activates mTORC2, glutamate dehydrogenase (GDH) and aspartate aminotransferase (ASAT) to catabolize excess glutamate in the hemolymph into α-KG, so as to maintain the TCA cycle and to support viral replication (56). In addition, α-KG can also be converted into isocitrate, and then used for the synthesis of lipids required by WSSV (57).

For the aromatic amino acids phenylalanine (Phe) and tyrosine (Tyr), Phe has been implicated in mammalian immune response (88), as it regulates T-cells proliferation and activation (201). Among aquatic animals, most of the studies involving the aromatic amino acids, especially Phe, has been in fish. For instance, Phe is reported to promote lysozyme expression in *Danio rerio*, acting as an essential amino acid for the elimination of resistant *Vibrio alginolyticus* (58), while in juvenile hybrid tilapia (*Oreochromis niloticus × Oreochromis aureus*), Phe levels affect immune performance (59). There are relatively few studies on Phe in crustaceans, which have mainly focused on the dietary requirements of Phe for optimal growth (202). Tyrosine hydroxylase, a key enzyme in Tyr metabolism, catalyzes the conversion of Tyr to L-DOPA, which then forms the catecholamines (dopamine, norepinephrine, and epinephrine) (203). Silencing of tyrosine hydroxylase has been shown to enhance the immunity of *P. vannamei*, even under low temperature stress (61, 62). Similarly, the catecholamine dopamine (DA) is reported to affect the immunity of crustaceans, as it suppresses the immune response of shrimp (*P. monodon* and *M. rosenbergii*) as well as increase their susceptibility to bacteria (*Lactococcus garvieae* and *Photobacterium damsela*) infection. DA receptors also couple with G protein to activate the cyclic adenylate (cAMP)-PKA, DAG-PKC or CaM pathway to regulate the immune system, and modulate the activities of antioxidant enzymes (63–65, 204). Thus, the enhancement of crustacean immunity after tyrosine hydroxylase silencing is achieved due to an inhibition of DA (or other catecholamines) synthesis.

Proline (Pro) is a key amino acid involved in protein synthesis (205) and an important regulator of metabolism (206), immunity (66), and together with its metabolite, hydroxyproline, play an important role in collagen synthesis and tissue repair (207). Proline metabolism plays an essential part in innate immune response, as infection of *Caenorhabditis elegans* with *P. aeruginosa*, results in the catabolism of Pro into P5C by proline dehydrogenase (PRODH), with the resultant P5C regulating ROS homeostasis and SKN-1 activation, to induce antibacterial response (208). Limited studies have explored the role of proline in crustaceans, but Pro is reported to play a role in the immune system of shrimp, where dietary Pro supplementation has been shown to improve the antioxidant and immune capacity of *P. vannamei* (66).

Recent studies have reported that histidine (His) plays an important role in the antioxidant capacity of fish. For instance, low His diets have been shown to inhibit the nuclear import of Nrf2 and to decrease the expression of antioxidant enzymes in juvenile *M. amblycephala* (67) and young *C. idella* (68). In crustaceans, the His metabolite, histamine, is reported to affect the immunity of *E. sinensis* by increasing the activities of PO and SOD, while decreasing the levels of THC, ACP, and AKP as well as the activities of intestinal digestive enzymes (69, 209).

Threonine (Thr) plays an important role in intestinal immunity (210), with high levels (3%) of dietary Thr shown to improve the intestinal tract of broilers and increase the IgA levels of ileum to enhance immune response (211). Deficiency of Thr is reported to induce decrease in lysozyme activity, and reduce the levels of C3, C4, and IgM as well as some anti-inflammatory factors, thereby impairing the growth and development of juvenile *C. idella* (70). Thr levels have also been reported to affect the growth performance of crustaceans. In juvenile *P. vannamei*, the SGR and protein efficiency ratio increased with dietary Thr supplementation, coupled with increased activity of SOD and PO (19, 212).

Dietary glycine (Gly) supplementation has been shown to increase WG and SRG of *P. vannamei* (213), while Gly and N-acetyl cysteine (NAC) supplementation improves the antioxidant capacity of *C. idella* (73). Alanine (Ala) has been implicated in the regulation of T-cells activation in mice (214) with no report in marine species. For the other amino acids such as serine (Ser), aspartate (Asp), and asparagine (Asn), there are currently no published reports on their role in crustacean immune-metabolic regulation. Despite the fact that there are currently no reported studies on the involvement of these amino acids and/or their metabolites in crustacean immunity, the importance of these amino acids in most organisms coupled with the fact that core metabolic pathways are conserved across species, it is plausible to conceive that these amino acids could also play vital roles in crustacean immune modulation. Further studies are needed in order to ascertain the involvement of these amino acids in immune-modulation in crustaceans.

## Conclusion and Future Perspectives

Amino acid metabolism is essential for maintaining normal growth and for generating metabolites important for physiological and pathophysiological processes in the body. The immune system, which is pivotal in protecting and defending the body from pathogens, is nourished and regulated by amino acids and their metabolites (215, 216). While some amino acids including cysteine, alanine, glutamine, etc., have been shown to directly affect the activation, proliferation, and differentiation of immune cells (88, 198, 214), key enzymes involved in amino acid metabolism have also been implicated as key regulators of the immune system (12, 208). Most of the research findings on the role played by amino acids and their metabolites in immunomodulation has been in mammals, with very few studies on crustaceans. Nevertheless, given that the core metabolic pathways are conserved across species, and the fact that exogenous supplementation of amino acids and amino acid metabolites have been shown to drastically improve the antioxidant capacity and immune parameters in fish and crustaceans, it indicates the importance of amino acids in immune-metabolic modulation in crustaceans. For instance, details are emerging on the pathogenicity and infection mechanism of WSSV, which requires glutamic acid for replication in shrimp (56). During Arg metabolism, increased activity of NOS has been shown to enhance functions of the immune system in *P. vannamei*, while Arg kinase promotes WSSV replication (24, 26). This suggests that Arg might be the bridge between energy metabolism and immune response.

In mammalian studies, Trp metabolites have been used as ligands to activate AhR (217), so as to regulate host intestinal immunity, which in turn, affects the composition of intestinal microbiota such as *Clostridium sporogenes*, *Ruminococcus gnavus*, *Lactobacillus*, *Clostridium*, *Bacteroides*, etc. (101). Similarly, Trp supplementation can enhance the immunity and antioxidant capacity of crustaceans, as well as increase intestinal microbiota (29, 30), which suggest that intestinal microbiota regulate the immune system of crustaceans *via* Trp metabolites. Trp metabolism may therefore reveal the relationship between crustaceans and microorganisms. In addition, studies in fish show that amino acids and their metabolites can regulate the immune system by activating immune related signaling pathways or enhance the synthesis of immune proteins. Since amino acids undergo similar metabolism in different species, it suggests that in crustaceans amino acids may also improve their immune response through similar mechanisms (**Figure 1**).

Crustacean farming has been hampered by several diseases and pathogens (218), as there is still limited understanding of their molecular immunology. Thus, to fully understand the immune response mechanisms of crustaceans, it is necessary to explore and delineate the key molecular factors such as amino acids and/or their metabolites that are involved in the modulation of these processes. Such insight would enable the institution of prudent and more effective disease control measures. Knowledge of immunomodulation by amino acids or their metabolites from similar studies in other animals such as fish could be inferred and possibly leveraged for useful application in crustacean aquaculture.

## Author Contributions

JA and YZ conceived the idea. ZH and JA performed the literature search, wrote, and revised the paper. YZ obtained funding and provided supervision. CZ, NT, YH, SL, and DF provided literature input and suggestions. All authors contributed to the article and approved the submitted version.

## Funding

This work was sponsored by National Natural Science Foundation of China (No.31872596), Department of Education of Guangdong Province (No.2017KZDXM033), and Science and Technology Planning Project of Guangdong Province (2017B020245001).

## Conflict of Interest

YH was employed by the company Guangdong Yuequn Marine Biological Research and Development Co., Ltd.

The remaining authors declare that the research was conducted in the absence of any commercial or financial relationships that could be construed as a potential conflict of interest.
